# Extracorporeal Membrane Oxygenation in Cardiogenic Shock due to Acute Myocardial Infarction: A Systematic Review

**DOI:** 10.1155/2020/6126534

**Published:** 2020-04-19

**Authors:** Marius Andrei Zavalichi, Ionut Nistor, Alina-Elena Nedelcu, Simona Daniela Zavalichi, Cătălina Marina Arsenescu Georgescu, Cristian Stătescu, Adrian Covic

**Affiliations:** ^1^Cardiovascular Diseases Institute “Prof. Dr. George I.M. Georgescu”, “Grigore T. Popa” University of Medicine and Pharmacy, Iasi, Romania; ^2^“C. I. Parhon” University Hospital, “Grigore T. Popa” University of Medicine and Pharmacy, Iasi, Romania; ^3^Research Methodology and Evidence Based Medicine Center, Iasi, Romania; ^4^Rehabilitation Clinical Hospital, “Grigore T. Popa” University of Medicine and Pharmacy, Iasi, Romania

## Abstract

**Background:**

Cardiogenic shock is associated with high mortality, despite new strategies for reperfusion therapy. Short-term circulatory support devices may provide adequate support for appropriate myocardial and organ perfusion.

**Objectives:**

This review is aimed at evaluating the impact on survival when using venoarterial extracorporeal membrane oxygenation (V-A ECMO) in patients with cardiogenic shock due to acute myocardial infarction (AMI).

**Methods:**

We performed a systematic review that included studies using V-A ECMO in patients with cardiogenic shock. Time on ECMO, side effects, and the number of deceased patients, transplanted or upgraded to durable assist devices were analysed. Literature search was done using PubMed/MEDLINE (inception (1969) to January 10, 2019), ProQuest (inception (January 14, 1988) to January 10, 2019), and clinicaltrials.gov (inception (September 12, 2005) to January 10, 2019), by 2 authors. This protocol is registered with PROSPERO (no. CRD42019123982).

**Results:**

We included 9 studies with a total of 1,998 adult patients receiving V-A ECMO for AMI-induced cardiogenic shock. Survival rate varied from 30.0% to 79.2% at discharge and from 23.2% to 36.1% at 12 months. Time on ECMO varied between 1.96 and 6.0 days. Reported serious adverse events were gastrointestinal bleeding (3.6%) and peripheral complications (8.5%).

**Conclusion:**

The use of V-A ECMO among patients with AMI-induced cardiogenic shock may provide survival benefits. However, V-A ECMO treatment effects are inconclusive because of limitations in cohort design and reporting.

## 1. Introduction

Myocardial infarction accounts for 5–10% of patients with cardiogenic shock [1, 2]. Additionally, cardiogenic shock represents the main cause of mortality in patients with acute myocardial infarction (AMI) [3], with the reporting data showing an increased incidence of cardiogenic shock from 6.5% in 2003 to 10.1% in 2010 [4].

Establishing blood flow by percutaneous coronary intervention (PCI) and a coronary artery bypass graft (CABG) remain the key approaches for patients with AMI. However, success rate remains low, despite maximum therapy [5].

New strategies for reperfusion therapy have been associated with improvement in survival rates, but significant disparities among trials may be observed [6].

The 2017 European Society of Cardiology Guidelines suggest the use of short-term active mechanical support in cardiogenic shock based on class IIb, level of evidence C [7].

The use of intra-aortic balloon counter pulsation (IABP) among patients with AMI and cardiogenic shock did not reduce early or late mortality, as demonstrated in the IABP-SHOCK II trial [8], while ventricular assist devices (VAD) and ECMO are increasingly popular but have not been sufficiently evaluated in clinical trials [7].

The extracorporeal membrane oxygenation (ECMO) machine provides support that resembles the cardiopulmonary bypass using a centrifugal pump and a membrane oxygenator with a drainage and return cannula. Venoarterial ECMO provides the benefit of maintaining an optimal cardiac output, before or after coronary revascularization, enabling the use of lower doses of vasoactive drugs. In some studies, it was associated with high survival rates (up to 51% survival to discharge) in cardiogenic shock, being used as rescue therapy in these patients [9], with short- and long-term survival benefits of cardiopulmonary resuscitation compared to standard care [10].

In addition, the Extracorporeal Life Support Organization guidelines include special algorithms for using ECMO as a bridge-to-recovery approach for postacute myocardial infarction [11].

Other mechanical devices such as Impella are used after ECMO commencement, to assure optimal haemodynamic conditions and reducing time on ECMO, playing a key role in cardiogenic shock [12].

Recent trials suggest the combination of V-A ECMO and the Impella device in the so-called ECPELLA strategy, providing more benefits than V-A ECMO with surgical venting, in order to avoid increased left ventricular afterload during extracorporeal support [13].

Despite such premises, survival benefits of ECMO therapy for cardiogenic shock are not consistent, covering a wide range of percentages that reflect a great variability of potential advantages versus disadvantages of this type of mechanical support [14].

This review is aimed at evaluating the impact on survival, potential benefits, and side effects of V-A ECMO in patients with cardiogenic shock after myocardial infarction (ST-segment elevation myocardial infarction and non-ST segment elevation myocardial infarction) in a systematic way.

## 2. Materials and Methods

### 2.1. Methods

#### 2.1.1. Protocol and Registration

This protocol has been registered in the PROSPERO database of systematic review protocols, under registration number CRD42019123982.

#### 2.1.2. Data Sources/Search Strategy

We have searched PubMed/MEDLINE (inception (1969) to January 10, 2019), ProQuest (inception (January 14, 1988) to January 10, 2019), and clinicaltrials.gov (inception (September 12, 2005) to January 10, 2019) without language restrictions. Hand searching for relevant articles was done on reference lists from textbooks, articles, and scientific proceedings. The search terms used and a detailed search strategy are shown in Table 1.

#### 2.1.3. Study Selection

We have searched for observational studies and randomized clinical trials for adults with myocardial infarction complicated by cardiogenic shock that were treated with ECMO for mechanical circulatory support and have reported data about the impact of V-A ECMO on survival, ECMO duration, complications associated with the use of ECMO (limb ischaemia, encephalopathy, acute kidney injury, infections, and bleeding), and the opportunity to switch to ventricular assist devices.

Studies that reported data for more than 10 patients were only included.

#### 2.1.4. Data Extraction and Synthesis

Data extraction was done independently by 2 authors (MZ and AN) using standardized data extraction forms. When more than one publication of a study was found, only the publication with the most complete data was included. Extracted data included identifiable information, study outcomes, details of the study protocol, and demographic data. We extracted the characteristics of each study, including type of ECMO; ECMO duration; survival rate at 1, 6, and 12 months; and if ECMO was used as a bridge to transplantation. Disagreements were resolved by consultation between all authors. Methods used were similar to the methods of Bilha et al. [15].

#### 2.1.5. Risk of Bias

Quality of the selected studies was independently evaluated by 2 reviewers (MZ and AN), using the Newcastle-Ottawa scale (NOS); according to the NOS, 3 methodological categories were used for assessment: selection (score 0-4), comparability (score 0-2), and outcome (score 0-3). Quality was considered high if the score was 7-9, intermediate if the score was 4-6, and low if the score was 0-3.

Disagreements were resolved by consensus [16].

### 2.2. Statistical Analysis

We performed a narrative synthesis using data extraction tables, independently carried out by 2 authors.

## 3. Results

**Figure 1:**
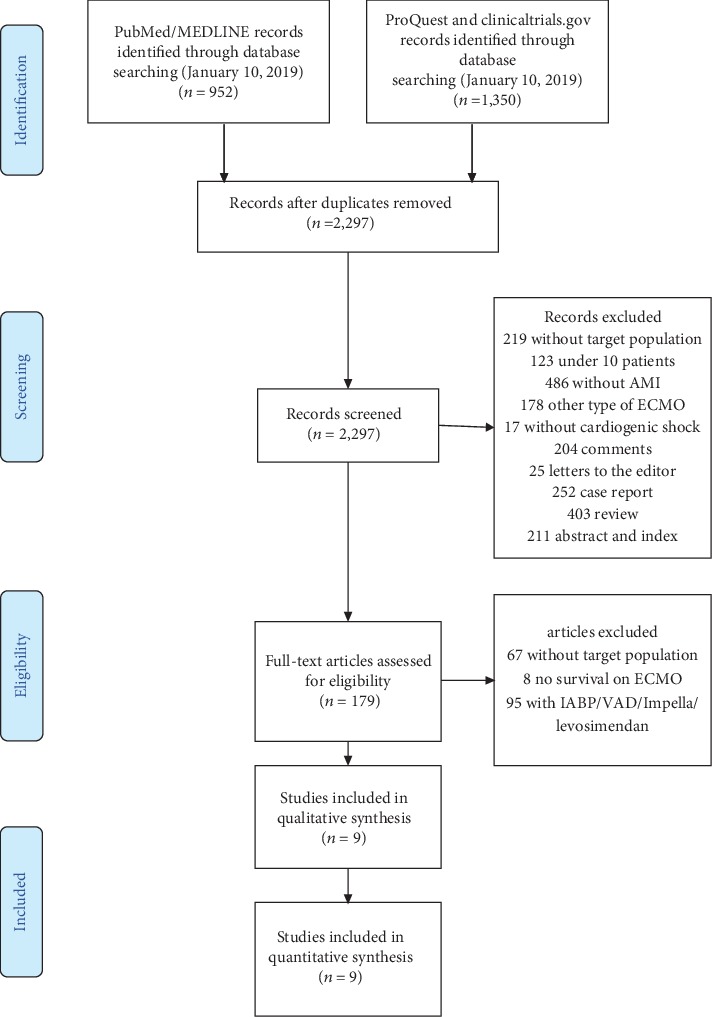
Selection process of the included studies.

For study selection, a flow diagram providing the selection process of the included studies is shown in Figure 1.

The initial search resulted in 2,302 potentially relevant articles. A thorough analysis of the abstracts led to the exclusion of 219 articles referring to several population categories that were of no interest for this review (children, pregnant women, and animal subjects); 681 articles were excluded because the outcomes were not reported (myocardial infarction/cardiogenic shock/V-A ECMO); 123 reported studies under 10 patients for analysis, as well as other case reports, editorials, and reviews (*n* = 1,095), were not included. Finally, 5 duplicates were also excluded.

A total of 179 full-text articles were thoroughly analysed; 8 of these were excluded due to absence of survival data, 67 did not include the target population, 95 were excluded because they reported data about the use of intra-aortic balloon counter-pulsation/percutaneous ventricular assist device prior to ECMO. After an in-depth analysis, 9 observational studies involving 1,998 patients were included in this systematic review.

### 3.1. Baseline Study and Patient Characteristics

The main characteristics of the included studies are presented in Table 2.

Median follow-up period generally varied between 1 and 12 months; 3 studies in the People's Republic of China, 2 in Taiwan, 1 in Germany, 2 in the United States of America, and 1 in South Korea were performed. The mean age of the patients involved varied between 55 and 65 years; males accounted for 76.02% of the total number of patients.

The most frequent comorbidities were diabetes mellitus and stroke. Negi et al. [17] have reported that 56.2% of the patients had diabetes. Stroke rates were similar, varying from 10.9% [18] to 14.2% [19]. Hypertension was present in 55.8% of the population included in the study by Chou et al. [20], in 46.6% of the patients by Chang et al. [18], and 45% by Huang et al. [21]. Several studies reported a history of previous heart disease [18–20, 22]. The comorbidities present in ECMO patients are summarized in Table 3.

### 3.2. ECMO Duration

ECMO duration varied: 1.96 days in Chang et al. [18], 2.75 days in Wu et al. [19], 5.0 days in Guenther et al. [23], 4.26 days in Huang et al. [21], and 6 days in Sandoval et al. [22] studies.

### 3.3. Survival on ECMO

#### 3.3.1. Survival Rate after Weaning from ECMO

Only 3 studies have reported the total number of patients weaned from ECMO and the number of those who did not survive to discharge after being weaned off [21–23]. The data is summarized in Table 4.

### 3.4. Study Outcomes

#### 3.4.1. V-A ECMO Survival at Discharge at 1, 6, and 12 Months

Overall, survival at discharge was reported in 8 out of the 9 studies included, with the highest rate registered by Sandoval et al. [22] (79.16%). Despite this survival rate, the number of patients included was low, with only 21 subjects and no follow-up data being available. Survival at 1 month after extracorporeal life support was 34%, reported by Chang et al. [18], 52% by Guenther et al. [23], 39.8% by Jeon et al. [24], and 58% by Negi et al. [17].

Survival at 6 months ranged from 33.6% in Wu et al. [19] to 37% in Jeon KH et al. [24].

Survival at 12 months was reported at 73% in Wu et al. [19], 23.2% in Chang et al. [18], 34.9% in Chou et al. [20], and 36.1% in Jeon et al. [24].

#### 3.4.2. Complications during Hospitalization of Patients with Cardiogenic Shock on ECMO Support

The most common adverse effect was acute kidney failure, seen in 45.7% in Wu et al. [19] (25.7% were patients with chronic kidney disease), 23% in Chang et al. [18] (8.8% already had chronic kidney disease), and 58.3% in a subgroup of Sattler et al. [25].

However, only these 3 studies reported data for acute kidney injury.

Considering peripheral complications, limb ischaemia was encountered in 8.5% of the study population [19].

In terms of cerebral complications, hypoxic ischaemic encephalopathy was the most common (75% in Huang et al. [21], 45.7% in Wu et al. [19]). Furthermore, ischaemic stroke and intracerebral haemorrhage were also found (2.8% and 1.7%, respectively, in Chang et al. [18]).

Gastrointestinal bleeding was reported by Chang et al. [18] in 63 patients, representing 3.6% (13.2% with previous gastric ulcer disease and 6.5% with cancer) of the study population.

Sepsis was found in 11.6% of patients by Chou et al. [20], with only 1 case of septic shock in Huang et al. [21]. Multiple organ failure was encountered in 48.8% in Chou et al. [20] and 39.1% in Guenther et al. [23] studies.

Data related to complications associated with the use of ECMO were not reported in 4 studies [17, 22, 24, 26]. The most frequent complications reported are summarized in Tables 2 and 5.

#### 3.4.3. Opportunity to Switch to Ventricular Assist Devices: Subgroup Analysis Transplantation and Assistive Devices

*(1) Heart Transplantation*. A total of 2 out of 1,998 patients included in this review were eligible to receive a heart transplant, after weaning from ECMO [19, 23].

*(2) Assistive Devices*. The usage rate of assistive devices was low, being reported by 2 studies. In the study conducted by Guenther et al. [23], 2 patients underwent biventricular assist device implantation (Berlin Heart EXCOR®) and 2 left ventricular assist device implantations (HeartWare®); 4 out of 24 patients (16.6%) enrolled in the study conducted by Sandoval et al. [22] were further placed on left ventricular assist devices.

*(3) Study Quality*. Quality score of the included studies ranged from 5 to 9, with a mean quality score of 7. This corresponds to a medium-to-high quality of the included studies. The detailed scores are provided in Table 6.

## 4. Discussion

### 4.1. Summary of Findings

This systematic review shows a great variability in survival rates for patients treated with ECMO. We have included 9 studies with 1,998 patients and showed that the use of ECMO might prove a useful tool to increase the survival rate in patients with cardiogenic shock due to myocardial infarction (which is less than 50% with only standard care [27]) (with rates varying from 30.0% to 76.2%). These highly heterogeneous data was partly due to the nature of observational studies included, with diverse populations. In fact, these rates are similar to the ones reported by the Extracorporeal Life Support Organization in 2017 with a survival-to-discharge rate of 41%, using V-A ECMO devices [28].

According to the 2017 European Society of Cardiology Guidelines for the management of acute myocardial infarction in patients presenting with ST-segment elevation, short-term mechanical circulatory support (ECMO) may be considered for patients with refractory shock (class IIb, level C). [7].

The use of ECMO in critical cases increased from 1.06 to 1.77 cases per 100,000 patients by 2014 in the USA and from 1.1 cases in 2007 to 6.2 cases in 2014 in Germany [29].

A crucial aspect that may improve future success is immediate use by multidisciplinary teams specialising in ECMO, facilitating the safest transportation to PCI/CABG centres. A prognostic tool for predicting survival may be the SAVE score created by Schmidt et al. [30], included in the 2016 European Society of Cardiology Guidelines for the Diagnosis and Treatment of Acute and Chronic Heart Failure [31].

Constant upgrade to durable solutions such as ventricular assist devices that ensure a bridge-to-survival or transplantation could become the cornerstone of modern cardiology. Extracorporeal life support followed by ventricular assist devices increases the chance of receiving a heart transplant, by gaining time to find the right donor [32].

Among patients with AMI-induced cardiogenic shock, the usage of V-A ECMO may provide benefits in terms of survival. However, treatment effects of V-A ECMO are inconclusive due to limitations in cohort methods and reporting [7].

In our opinion, complications such as multiple organ failure, cerebral complications, and kidney failure may be related to cardiogenic shock, rather than to the use of ECMO.

ECMO was mostly associated with acute kidney failure (with a high percentage of patients having previous renal impairment), being a common complication, as it is shown in a systematic review that included 46 studies performed in patients treated with ECMO, where the occurrence rate was 52% [33].

In our study, rates varied between 24% and 47%, a complication that may be prevented by reducing the time to insertion of V-A ECMO. Additionally, ECMO infection prevention may be achieved by performing an accurate procedure. Nonetheless, vascular complications such as haemorrhage and limb ischaemia, seen in 8.5% of the included patients in our study, had similar rates, as reported in the literature.

## 5. Conclusions

Our study has its limitations and strengths. We performed a systematic literature search and a detailed survival analysis. However, our study could only identify observational studies. Additionally, sample size was relatively small, and data were not fully reported.

We could not exclude publication bias of original studies, as authors who did not register positive results on ECMO, or did not find any effect at all, were less likely to publish their results.

V-A ECMO for patients with acute myocardial infarction-induced cardiogenic shock represents a temporary support that provides benefits compared to standards of care, being an upgradable device for advanced life support that could assure a higher survival rate.

## Figures and Tables

**Table 1:** Keywords used for search strategy.

Keywords
Extracorporeal membrane oxygenation
ECMO
Veno-arterial extracorporeal membrane oxygenation
Veno-arterial ECMO
V-A ECMO
Mechanical circulatory support
Extracorporeal life support
ECLS
Cardiogenic shock
CS
Acute myocardial infarction
AMI

^∗^ECLS: extracorporeal life support; CS: cardiogenic shock; AMI: acute myocardial infarction.

**Table 2:** Baseline and patient characteristics.

Study	ECMO duration	Survive-to-discharge (no. of patients)	Survival rate at 1 month, 6 months, and 12 months	ECMO complications	Transplant	Assist device
Negi et al. [17]	NA	9 (58%)	58% 1 month NA 6 months NA 12 months	NA	NA	NA
Chang et al. [18]	1.96 days	576 (33.8%)	34% 1 month NA 6 months 23.2% 12 months	Acute renal failure *n* = 393 Gastrointestinal bleeding *n* = 63 Intracerebral haemorrhage *n* = 29 Ischaemic stroke *n* = 49	NA	NA
Wu et al. [19]	66 h for PCI (2-259) 100 h for CABG (43-504)	14 (40%)	NA 1 month 84% 6 months (of survivors) 73% 12 months (of survivors)	Limb ischaemia *n* = 3 Major anoxic encephalopathy *n* = 16 Acute renal failure *n* = 16	1	0
Chou et al. [20]	NA	15 (34.9%)	NA 1 month NA 6 months 34.9% 12 months	MOF *n* = 21 Sepsis *n* = 5 Other (ischaemic bowel disease, brain death) *n* = 2	NA	NA
Huang et al. [21]	102.3 ± 66.6 h	6 (30%)	NA 1 month NA 6 months NA 12 months	Septic shock *n* = 1 MOF *n* = 1 Hypoxic ischaemic encephalopathy *n* = 15 Coagulation dysfunction *n* = 12	NA	NA
Sandoval Y et al. [22]	6 (4-7) days	19 (79.16%)	79.16% 1 month NA 6 months NA 12 months	NA	0	4
Guenther et al. [23]	120 ± 81 h	NA	52% 1 month NA 6 months NA 12months	MOF *n* = 9 Diffuse encephalopathy *n* = 1 Intermittent haemodialysis *n* = 1	1	4
Jeon et al. [24]	NA	43 (39.8%)	39.8% 1 month 37% 6 months 36.1% 12months	NA	NA	NA
Fu et al. [26]	NA	12 (44%)	NA 1 month NA 6 months NA 12 months	NA	NA	NA

∗h: hours; CABG: coronary artery bypass graft; MOF: multiple organ failure.

**Table 3:** Comorbidities associated with the use of ECMO.

Study	Year	Country	Design	Year of follow-up	Follow-up	N	Type of ECMO	Age (years)	Gender	Comorbidities
Negi et al. [17]	20 15	USA	Retrospective	NA	NA	16	V-A ECMO	NA	NA	Diabetes *n* = 9
Chang et al. [18]	20 16	Taiwan	Retrospective	12.2002-12.2012	2.89 months	17 05	V-A ECMO	57.29 ± 16.1 y	70.6% male	Diabetes *n* = 533 Hypertension *n* = 796 Cerebral vascular disease *n* = 186 Cardiovascular disease *n* = 1106 Chronic kidney disease *n* = 151 Chronic liver disease *n* = 151 Hyperlipidaemia *n* = 455 Cancer *n* = 111 Gastric ulcer *n* = 226
Wu et al. [19]	20 13	China	Retrospective	06.2003-12.2011	12 months (169)	35	V-A ECMO	65.5 y	77.5% male	Diabetes mellitus *n* = 15 Serum creatinine > 2 mg/dl *n* = 7 Chronic dialysis *n* = 2 Previous PCI *n* = 6 Previous AMI *n* = 9 Previous stroke *n* = 5
Chou et al. [20]	2013	Taiwan	Retrospective	01.2006- 07.2010	12 mo	43	V-A ECMO	60.525 y	93%	Hypertension *n* = 24 Diabetes *n* = 15 Stroke *n* = 6 Chronic lung insufficiency *n* = 1 Previous heart disease *n* = 15
Huang et al. [21]	2017	China	Retrospective	01.2009–01.2015	30 days	20	V-A ECMO	58.8 ± 13.9 y	85% male	Hypertension *n* = 9 Diabetes *n* = 6 Old myocardial infarction *n* = 7 Active smoke *n* = 15
Sando val et al. [22]	2015	USA	Retrospective	NA	1 mo	21	V-A ECMO	62 y	62% male	History of coronary artery disease *n* = 5 Prior myocardial infarction *n* = 3 Diabetes *n* = 2
Guent her et al. [23]	2013	Germany	Retrospective	02.2012-08.2013	30 days	23	V-A ECMO	55 ± 14 y	15% female	NA
Jeon et al. [24]	2018	South Korea	Retrospective	01.2006-12.2016	12 mo	108	V-A ECMO	64.95 ± 11.1 y	NA	NA
Fu HX et al. [26]	2017	China	Retrospective	01.2014- 03.2017	NA	27	V-A ECMO	NA	NA	NA

∗V-A ECMO: venoarterial extracorporeal membrane oxygenation; PCI: percutaneous coronary intervention; AMI: acute myocardial infarction.

**Table 4:** Patients weaned from ECMO.

Study	Number of patients weaned from V-A ECMO	Number of nonsurvivors after weaning
Negi et al. [17]	NA	NA
Chang et al. [18]	NA	NA
Wu et al. [19]	22	NA
Chou et al. [20]	15	NA
Huang et al. [21]	8	2
Sandoval et al. [22]	16	5
Guenther et al. [23]	15	3
Jeon et al. [24]	NA	NA
Fu et al. [26]	NA	NA

**Table 5:** Most frequent complications associated with the use of ECMO.

Outcome	Number of studies	Number of events
Limb ischaemia	1	3
Encephalopathy	3	32
Ischaemic stroke	1	49
Intracerebral haemorrhage	1	29
Gastrointestinal bleeding	1	63
Acute renal failure	3	410
MOF	3	31
Sepsis	2	6

^∗^MOF: multiple organ failure.

**Table 6:** Newcastle-Ottawa scale for assessment of quality of included studies (each asterisk represents if individual criterion within the subsection was fulfilled).

Quality assessment Criteria	Acceptable (^∗^)	Wu et al.	Chang et al.	Guenther et al.	Fu et al.	Huang et al.	Chou et al.	Sandoval et al.	Jeon et al.	Negi et al.
(1) Representativeness of the exposed cohort	Representative of average adult in community (age/sex/being at risk of disease)	^∗^	^∗^	^∗^	^∗^	^∗^	^∗^	^∗^	^∗^	^∗^
(2) Selection of the nonexposed cohort	Drawn from the same community as the exposed cohort	^∗^	^∗^	—	—	—	^∗^	—	—	—
(3) Ascertainment of exposure	Secure record, structured interview	^∗^	^∗^	^∗^	^∗^	^∗^	^∗^	^∗^	^∗^	^∗^
(4) Demonstration that outcome of interest was not present at the start of the study		^∗^	^∗^	^∗^	^∗^	^∗^	^∗^	^∗^	^∗^	^∗^
(5) Adequate control for the most important confounder?		^∗^	^∗^	^∗^	—	^∗^	—	^∗^	—	^∗^
(6) Adequate control for any additional factor?		^∗^	^∗^	^∗^	—	^∗^	^∗^	—	—	—
(7) Assessment of outcome	Independent or blind assessment	^∗^	^∗^	^∗^	^∗^	^∗^	^∗^	^∗^	^∗^	^∗^
(8) Was follow-up long enough for outcomes to occur?		^∗^	^∗^	^∗^	^∗^	—	^∗^	^∗^	^∗^	^∗^
(9) Adequacy of follow-up of cohorts	Complete follow-up, or subjects lost to follow-up unlikely to introduce bias	^∗^	^∗^	^∗^	—	—	^∗^	^∗^	^∗^	^∗^
Overall quality score (maximum = 9)		9	9	8	5	6	8	7	6	7

## Data Availability

The data supporting this Systematic review are from previously reported studies and datasets, which have been cited. The processed data are available from the corresponding author upon request.
